# Gastrointestinal flora and serum metabolomic elucidation of *Astragali Radix* water decoction intervention in subclinical bovine mastitis

**DOI:** 10.3389/fvets.2025.1611467

**Published:** 2025-08-18

**Authors:** Jianpeng Yan, Ke Zhou, Ting Ma, Peng Ji, Yanming Wei

**Affiliations:** ^1^Tranditional Chinese Veterinary Medicine Laboratory, College of Veterinary Medicine, Gansu Agricultural University, Lanzhou, China; ^2^Lanzhou Center for Animal Disease Prevention & Control, Lanzhou, China

**Keywords:** Astragali Radix, subclinical bovine mastitis, phytochemical untargeted metabolomics, metabolomics, 16S rRNA

## Abstract

**Background:**

This study addresses the global challenge of subclinical bovine mastitis (SCBM) in dairy cows, a prevalent disease causing substantial economic losses, by investigating the mechanistic basis of *Astragali Radix*, a traditional herbal remedy with empirically validated efficacy but incompletely understood modes of action.

**Methods:**

Initially, the active components of *Astragali Radix* were identified using LC-MS/MS. Dose-response trials were conducted in Holstein cows (*n* = 24 SCBM cases; *n* = 6 healthy controls), along with multi-omics integration, including 16S rRNA sequencing for rumen/feces microbiota and UHPLC-MS metabolomics for serum analysis. The therapeutic effects of Astragali Radix water decoction (ARWD) on milk production, inflammatory markers, immune parameters, and oxidative stress were systematically evaluated.

**Results:**

ARWD administration dose-dependently improved milk yield and protein content while reducing somatic cell counts. Serum pro-inflammatory cytokines (TNF-α, IL-6, IL-1β) decreased, contrasting with increases in immunoglobulins (IgA, IgM, IgG) and enhanced superoxide dismutase activity. Microbiota restructuring featured ruminal enrichment of Bifidobacterium and fecal dominance of Rikenellaceae_RC9_gut_group, coupled with suppression of pro-inflammatory taxa (e.g., Christensenellaceae_R-7_group). Metabolomic analysis identified four ARWD-responsive biomarkers, notably Spirotaccagenin and Pelanin, operating through linoleic acid metabolism and phospholipase D signaling pathways. Strong correlations linked microbial shifts to improved lactation parameters and reduced inflammation.

**Conclusion:**

The findings establish that ARWD alleviates SCBM through coordinated microbiota remodeling and metabolic reprogramming, specifically enhancing antioxidant defenses, restoring mammary barrier integrity, and modulating immune-inflammation crosstalk, with optimal efficacy at 0.4 g·kg^−1^·d^−1^ dosage. This mechanistic validation positions ARWD as a scientifically grounded, eco-friendly alternative for sustainable mastitis management, reconciling therapeutic effectiveness with agricultural economic priorities.

## 1 Introduction

In recent years, subclinical bovine mastitis (SCBM) has emerged as a significant issue in the dairy industry, second only to clinical mastitis. This condition has a profound impact on milk yield and quality, negatively affecting overall herd health and ultimately reducing the profitability of dairy farms (1). Veterinary experts agree that controlling somatic cell count (SCC) is crucial for sustainable dairy production. SCBM is characterized by the gradual onset, high contagion rates, and increased SCC levels in milk, often going unnoticed until it causes considerable economic losses (2). Therefore, it is vital to implement preventive measures during the SCBM phase to ensure the health of bovine mammary glands. While traditional antibiotic treatments are commonly employed, they can adversely affect milk quality and pose risks to human health due to the potential for antimicrobial resistance, highlighting the urgent need for sustainable alternatives (3).

According to traditional Chinese veterinary medicine (TCVM), deficiencies in qi and blood contribute to the development of SCBM, increasing cows' susceptibility to bacterial colonization in the mammary tissues. Key pathogens involved include *Staphylococcus aureus* and *Streptococcus agalactiae*, which take advantage of weakened immune states to establish persistent infections (4).

The mammary gland serves as an essential component of the immune system, employing intricate mechanisms to protect against bacterial infections, which are vital for managing infections. Recent research indicates that the gastrointestinal microbiota, often called the “second genome,” significantly contributes to the immune defenses of the mammary gland through interactions along the gut-mammary axis (5). This microbial community is crucial for the immune system, particularly in identifying pathogens within the mammary glands and regulating inflammation.

The gut-immune axis has emerged as a significant area of research, with compelling evidence indicating a bidirectional communication between gut microbiota and host immunity (6). An imbalance in gut microbiota is linked to various health problems, including infections and inflammatory diseases. In dairy cows, changes in gut microbiota composition can heighten the risk of mastitis, even in subclinical cases (7). This microbial dysregulation impacts the production of immunomodulatory metabolites, such as short-chain fatty acids (SCFAs), which plays a crucial role in regulating the immune system throughout the body (8). In this regard, herbal medicines have shown promising immunomodulatory properties without adverse effects. For instance, *Astragali Radix*, a prominent herb known for boosting qi, has been found to modulate immune responses and affect gut microbiota. Experimental studies indicate that this herb promotes the growth of beneficial bacteria like *Lactobacillus* and *Bifidobacterium* while inhibiting harmful pathogens such as *Escherichia* and *Salmonella* (9). Additionally, bioactive compounds found in Astragalus, including polysaccharides and saponins, significantly enhance macrophage phagocytosis, promote the maturation of dendritic cells, and stimulate T-lymphocyte proliferation (10). Moreover, advanced technologies like high-throughput 16S rRNA gene sequencing and metabolomics are shedding light on the interactions between traditional Chinese medicine (TCM), gut microbiota, and immune function. By influencing microbial communities and regulating metabolic pathways, these innovative methods help clarify the mechanisms underlying TCM interventions (11, 12).

This study explored the therapeutic effectiveness and underlying mechanisms of *Astragali Radix* water decoction (ARWD) in treating bovine SCBM by utilizing fecal 16S rRNA sequencing and serum untargeted metabolomics. Additionally, the research identified the bioactive components of ARWD decoction through LC-MS/MS analysis. The findings provide a scientific foundation for clinical application of ARWD in SCBM prevention and control within veterinary practice.

## 2 Materials and methods

### 2.1 Materials and reagents

*Astragali Radix* was purchased from Lanzhou Yellow River medicine market. Origin: Liupanshan Region, China. The following kits were used in this study: malondialdehyde (MDA) test kit (catalog No. YJ016824), superoxide dismutase (SOD) test kit (catalog No. YJ036559), myeloperoxidase (MPO) test kit (catalog No. YJ300741), lactate dehydrogenase (LDH) test kit (catalog No. YJ520026), immunoglobulin A (IgA) ELISA kit (catalog No. YJ542063), immunoglobulin G (IgG) ELISA kit (catalog No. YJ330698), immunoglobulin M (IgM) ELISA kit (catalog No. YJ627279), Interleukin-2 (IL-2) ELISA kit (catalog No. YJ002498), interleukin-1 β (IL-1 β) ELISA kit (catalog No. YJ064295), interleukin-6 (IL-6) ELISA kit (catalog No. YJ064296) and tumor necrosis factor α (TNF- α) ELISA kit (catalog No. YJ077389), all the above were purchased from Shanghai Meilian Biotechnology Company.

### 2.2 Preparation of ARWD

*Astragali Radix* were mixed with distilled water at a 1:10 (w/v) ratio. The mixture was vigorously boiled, then simmered at low heat for 30 min and filtered through four-layer sterile gauze. The residue underwent re-extraction with an 8-fold volume of distilled water following identical boiling/simmering conditions, followed by gauze filtration. Both filtrates were combined for subsequent experiments.

### 2.3 Experimental animals and grouping

All experimental cows were obtained from the Gansu Holstein Dairy Cattle Breeding Center and selected as multiparous, mid-lactation individuals (3–9 years old) with comparable body weights. The animals were fed mixed ration (TMR) three times a day at 8:00, 16:00, and 21.30 respectively (Table 1). Mammary health was assessed via SCC and clinical mastitis evaluation. Based on established criteria (13, 14), cows with SCC < 200,000 cells/ml were considered healthy, while those with SCC > 200,000 cells/ml without clinical symptoms were diagnosed with SCBM. Untreated positive cows with SCBM for 3–5 days (*n* = 24) were randomly assigned to four experimental groups (*n* = 6 per group): *Astragali Radix* water decoction High-dose group (0.4 g·kg^−1^·d^−1^, AR_H), *Astragali Radix* water decoction Medium-dose group (0.2 g·kg^−1^·d^−1^, AR_M), *Astragali Radix* water decoction Low-dose group (0.1 g·kg^−1^·d^−1^, AR_L). Model group: untreated SCBM controls (MOD). Six additional healthy cows received equivalent volumes of water as negative controls (NC). All ARWD treatments were administered orally via force-feeding for seven consecutive days. Sample Collection: 5 ml of blood were collected from each cow through the tail vein 1 h after feeding on the morning of day 8. The blood samples were then centrifuged at 3,000 r/min for 15 min at 4°C to separate the serum. Rumen fluid was extracted by inserting a rumen sampler via the mouth into the rumen and using a syringe. The first two tubes of rumen fluid were discarded to prevent salivary contamination. Approximately 150 ml of rumen fluid was sampled from each cow. Fecal samples were collected from the rectum using sterile long-arm gloves, 3 h after feeding, and placed in sterile, sealed plastic bags. Fecal and rumen fluid samples were immediately snap frozen in liquid nitrogen and stored at −80°C. Milk samples were collected and transported on ice for somatic cell count (SCC) analysis and milk composition testing.

**Table 1:** Chemical composition of TMR.

**Ingredient**	**Content (%)**	**Nutrient composition**	**Content (%)**
Corn silage	56.5	Dry matter	47.4
Brewing grain	14.6	Neutral detergent fiber	31.1
Alfalfa hey	2.4	Crude protein	16.5
Oat grass	1.6	Ether extract	3.5
Concentrate feed	24.9	rumen undegradable protein	33.4

The concentrate feed is composed of corn (53.2%), soybean meal (32.9%), cottonseed (5%), fat (2.2%), salt (0.8%), and premix (5.9%).

### 2.4 LC-MS/MS analysis of ARWD samples

The LC-MS/MS analysis was performed using a UHPLC-Q Exactive system (Thermo Scientific) equipped with a UPLC BEH C18 column (2.1 × 100 mm i.d., 1.7 μm). The mobile phase consisted of (A) 2% acetonitrile containing 0.1% formic acid and (B) acetonitrile with 0.1% formic acid. Full-scan MS data were acquired in both positive and negative ionization modes over a mass range of 70–1,050 m/z at a resolution of 70,000. Data processing, including peak alignment, extraction, and quantification, was conducted using Compound Discoverer QI v3.0 (WatersCorporation, Milford, USA) software. Metabolite identification was achieved by matching accurate mass and MS/MS spectra against the Majorbio Bio-Pharm Technology Co., Ltd (Shanghai, China) database with (mass accuracy threshold of <10 ppm).

### 2.5 Milk yield statistical analysis

Milking was performed using rotary milking parlors pre- and post-treatment, with individual milk yields recorded for each cow.

### 2.6 Somatic cell count and milk composition analysis

SCC and milk composition parameters [fat, protein, lactose, total milk solids (TS), milk urea nitrogen (MUN)] were analyzed using a CombiFoss™ 7 analyzer (Foss Analytical, Denmark).

### 2.7 Detection of serum oxidative stress markers

The biochemical test kit was used to measure the levels of LDH, MPO, MDA, and SOD in serum. All experimental procedures were strictly conducted according to the manufacturer's instructions for the reagents.

### 2.8 Detection of serum inflammatory cytokines

The levels of IL-1β, IL-6, IL-2, and TNF-α in serum were measured using ELISA test kits. All experimental procedures were strictly carried out according to the instructions provided by the manufacturer for the reagents.

### 2.9 Detection of serum immunoglobulin

The levels of IgA, IgM, and IgG in serum were measured using biochemical test kits. All experimental procedures were strictly followed according to the instructions provided by the manufacturer for the reagents.

### 2.10 Rumen and fecal microbiota analysis

After drug administration, rumen fluid and rectal content were collected and stored at −80°C. According to the manufacturer's instructions, total microbial genomic DNA was extracted from 18 gastric juice samples and 18 fecal samples using the E.Z.N.A.^®^ soil DNA Kit (Omega Bio-tek, Norcross, GA, U.S.). The mass and concentration of DNA were determined by 1.0% agarose gel electrophoresis and NanoDrop2000 spectrophotometer (Thermo Scientific, United States), and were stored at −80°C for further use. The hypervariable region V3–V4 of the bacterial 16S rRNA gene were amplified with primer pairs 338F (5′-ACTCCTACGGGAGGCAGCAG3′) and 806R (5′-GGACTACHVGGGTWTCTAAT3′) by T100 Thermal Cycler PCR thermocycler (BIO-RAD, USA). The PCR reaction mixture included 4 μl of 5 × Fast Pfu buffer, 2 μl of 2.5 mm dNTPs, 0.8 μl (5 μm) for each primer, 0.4 μl of Fast Pfu polymerase, 10 ng of template DNA, and ddH2O up to a final volume of 20 μl. The PCR amplification cycle conditions are as follows: initial denaturation at 95°C for 3 min, denaturation at 95°C for 30 s, annealing at 55°C for 30 s, extension at 72°C for 45 s, single extension at 72°C for 10 min, and conclusion of 27 cycles at 4°C. The PCR product was extracted from 2% agarose gel and purified using the PCR Clean-Up Kit (Yuhua, Shanghai, China) according to manufacturer's instructions and quantified using Qubit 4.0 (Thermo Fisher Scientific, USA), and the purified amplifiers were aggregated in equal molar amounts. 2 × 300 bp paired-end sequencing was performed on the Illumina Nextseq2000 platform (Illumina, San Diego, USA) according to the standard protocols by Majorbio Bio-Pharm Technology Co., Ltd. (Shanghai, China).

### 2.11 Serum untargeted metabolomics analysis

Serum samples from blank control, model, and astragalus intervention groups (*n* = 6/group) were extracted with methanol:acetonitrile (1:1, v/v). After ultrasonication (5°C, 40 kHz, 30 min) and incubation (−20°C, 30 min), supernatants were collected by centrifugation (13,000 g, 4°C, 15 min), dried under nitrogen, and reconstituted in acetonitrile:water (1:1, v/v). Quality control (QC) samples were processed identically.

Metabolites were analyzed using UHPLC-Q Exactive Focus MS (Thermo Fisher) with an ACQUITY UPLC HSS T3 column (100 × 2.1 mm, 1.8 μm). Mobile phases: (A) 95% water/5% acetonitrile (0.1% formic acid); (B) 47.5% acetonitrile/47.5% isopropanol/5% water (0.1% formic acid). Flow rate: 0.40 ml/min, injection volume: 5 μl, column temperature: 40°C. MS parameters: ±3.50 kV spray voltage, 325°C capillary temperature, full scan at 81–1,000 m/z (70,000 resolution), HCD fragmentation (30 eV).

Raw data were processed in Progenesis QI v3.0 for peak alignment. Metabolites were identified by matching MS/MS spectra against HMDB, Metlin, and Majorbio Bio-Pharm Technology Co., Ltd. (Shanghai, China) in-house databases (MS error < 10 ppm, spectral score filtering). Differential metabolites underwent pathway enrichment analysis (*P* < 0.05).

### 2.12 Correlation analysis

Spearman's rank correlation analysis was performed to investigate relationships between differential metabolites and gut microbiota among NC, MOD, and AR_H groups. Additionally, pairwise correlations were analyzed between metabolites/gastrointestinal microbiota and dairy parameters (SCC, milk yield, milk composition), inflammatory factors, immune/antioxidant indices.

### 2.13 Statistical analysis

Gastrointestinal flora alpha diversity was statistically determined using the Kruskal–Wallis test, and beta-diversity was statistically determined using the ANOSIM test. Statistical analyses were performed using one-way ANOVA in GraphPad Prism 8 (GraphPad Software), with significance levels defined as *P* < 0.05 (significant), *P* < 0.01 (highly significant), and *P* > 0.05 (not significant).

## 3 Results

### 3.1 LC-MS/MS analysis of ARWD

**Figure 1:**
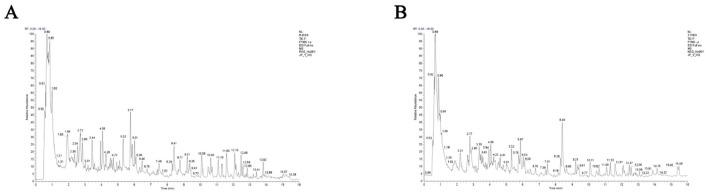
LC-MS/MS total ion chromatograms of ARWD in positive and negative ion modes. **(A)** Positive ion mode (POS); **(B)** negative ion mode (NEG).

Total ion chromatograms were acquired in both positive and negative ion modes. As shown in Figure 1, well-resolved peaks with uniform distribution were observed under the current analytical conditions. Qualitative analysis was performed by matching the mass spectrometry data matrix (retention time, m/z, and peak intensity) against the MJBIOTCM database. Nine compounds were identified, including flavonoids, steroids, and their derivatives (Table 2).

**Table 2:** Identification results of Astragalus samples by LC-MS/MS analysis.

**Item**	**Compound name**	**Retention time (min)**	**Y_HQ**	**[M/Z]**	**Error (ppm)**
1	Astragaloside III	9.3573	1,824, 771.43	802.4935	−1.5223
2	Astragaloside VI	8.6389	1,118, 351.30	964.5454	−2.2808
3	Ononin	5.3179	25,164, 078.81	431.1329	−1.7787
4	Calycosin-7-O-β-D-glucoside	4.0636	9,787, 917.63	491.1199	0.8519
5	Astragaloside II	10.6241	12,801, 642.07	871.4708	1.3701
6	Calycosin	5.8688	14,925, 299.13	283.0612	−0.0517
7	Astragaloside IV	8.2492	132, 954.70	829.4596	0.6632
8	Formononetin	8.4049	19,202, 627.74	267.0663	−0.1162
9	Isoastragaloside IV	9.3646	745, 646.04	819.4311	1.0494

### 3.2 Effects of ARWD on milk yield in SCBM cows

**Figure 2:**
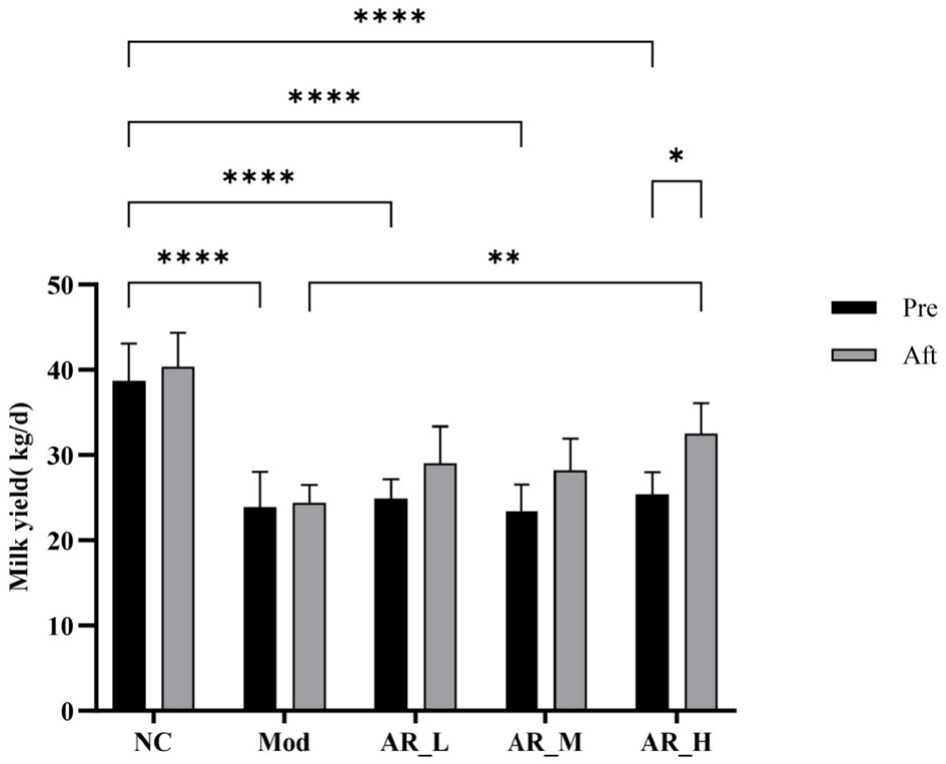
Milk yield changes. Differences between the two groups are indicated by (^*^), ^*^P < 0.05, ^**^P < 0.01, ^***^P < 0.001, ^****^P < 0.0001.

Analysis of milk yield before and after oral administrations revealed significant intergroup differences. Pretreatment milk yield in the MOD group was significantly lower than the NC group (*P* < 0.01). Post-intervention, all ARWD treated groups (AR_L, AR_M, AR_H) exhibited increased milk yields compared to baseline levels. Notably, the AR_H group showed marked improvement vs. the MOD group (*P* < 0.01), approaching NC group values. AR_L and AR_M groups demonstrated moderate milk yield increases post-treatment, though these changes lacked statistical significance (*P* > 0.05) (Figure 2).

### 3.3 Effects of ARWD on SCC and milk composition in SCBM cows

**Figure 3:**
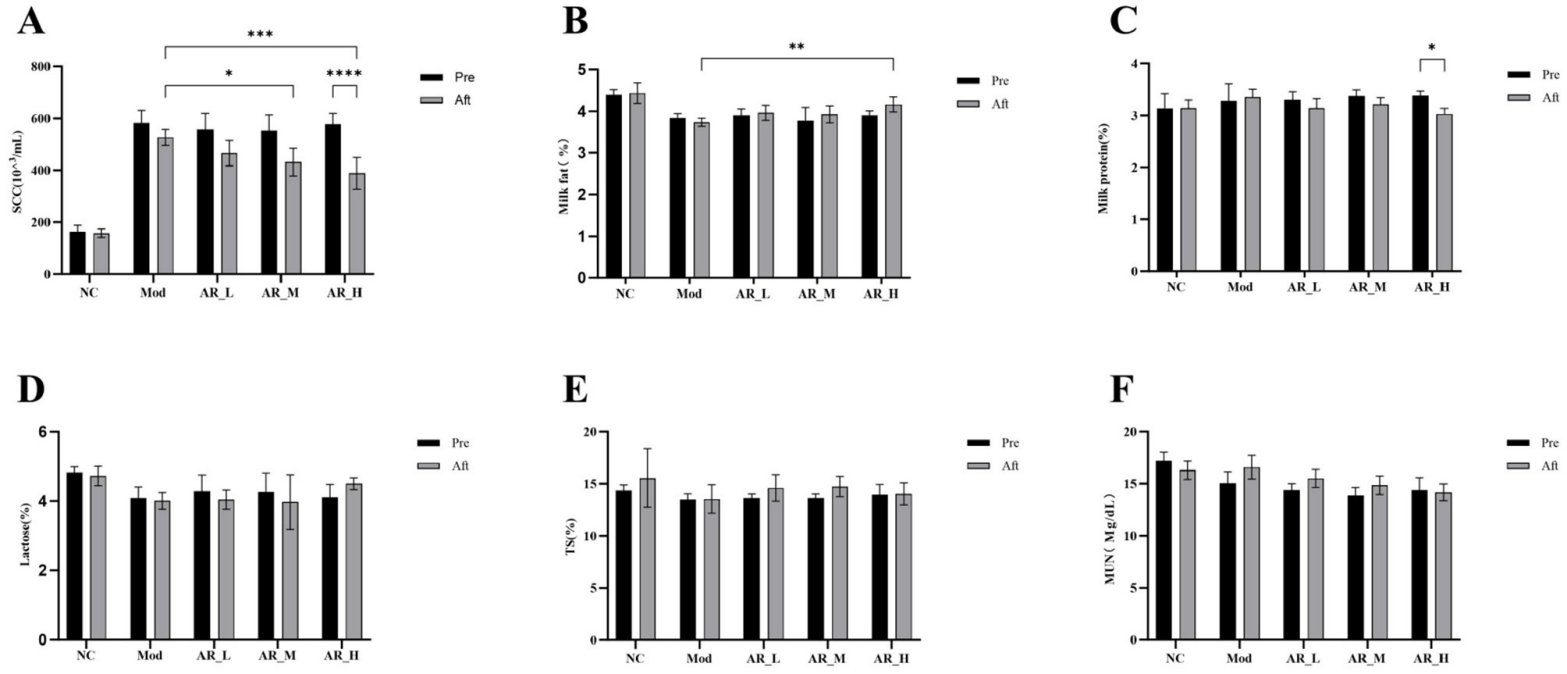
Changes of the somatic cells and milk components. **(A)** SCC; **(B)** milk fat; **(C)** milk protein; **(D)** latctose; **(E)** TS; **(F)** MUN. The differences between the two groups are indicated by (^*^), ^*^P < 0.05, ^**^P < 0.01, ^***^P < 0.001, ^****^P < 0.0001.

Post-treatment SCC analysis demonstrated significant reduction in the AR_H group vs. baseline (*P* < 0.01) and MOD group (*P* < 0.01). The AR_M group showed moderate SCC decrease compared to MOD (*P* < 0.05), while AR_L exhibited no significant change (Figure 3A).

Milk fat content analysis showed that after ARWD intervention, the fat content in the AR_H group was significantly higher than that in the MOD group (*P* < 0.01). Compared with before treatment, the fat content in the AR_H, AR_M, and AR_L groups increased after ARWD intervention, but the increase was not significant (Figure 3B).

Milk protein content analysis indicated that the protein levels in the AR_H group after treatment were significantly lower than before treatment (*P* < 0.05). Compared with the MOD group, the protein contents in the AR_H, AR_M, and AR_L groups were all lower than those in the MOD group and lower than before administration (Figure 3C).

Lactose, Total Solids, and Milk Urea Nitrogen analysis revealed that no statistically significant differences in lactose, total solids (TS), or milk urea nitrogen (MUN) were detected among experimental groups relative to NC (*P* > 0.05). Slight increases in lactose and TS were observed in AR_H and AR_M groups, though these trends did not reach statistical significance (Figures 3D–F).

### 3.4 Effects of ARWD on serum inflammatory cytokines in SCBM cows

**Figure 4:**
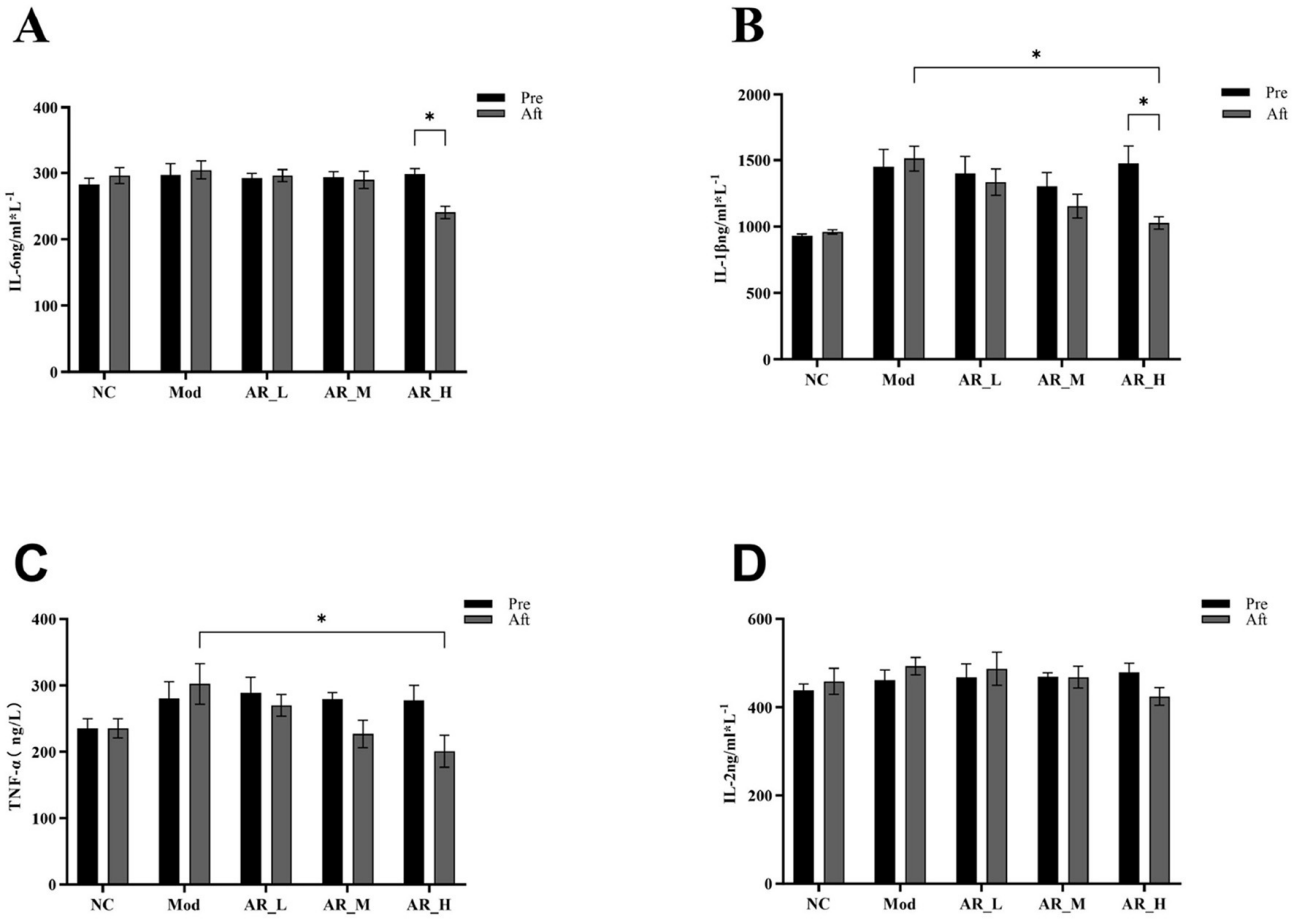
Changes of the serum inflammatory cytokines. **(A)** IL-6; **(B)** IL-1β; **(C)** TNF-α; **(D)** IL-2. The differences between the two groups are indicated by (^*^), ^*^P < 0.05.

As shown in Figure 4, IL-6 expression in the AR_H group was significantly reduced post-intervention compared to pre-treatment (*P* < 0.01). Similarly, IL-1β levels showed marked reduction in AR_H vs. both pre-treatment (*P* < 0.05) and MOD group (*P* < 0.05). TNF-α expression in AR_H group was significantly lower than MOD group (*P* < 0.05). In contrast, AR_M and AR_L groups exhibited no significant alterations in IL-6, IL-1β, or TNF-α levels compared to NC or MOD groups (*P* > 0.05). IL-2 expression remained unchanged across all experimental phases (Figure 4).

### 3.5 Effects of ARWD on oxidative stress in SCBM cows

**Figure 5:**
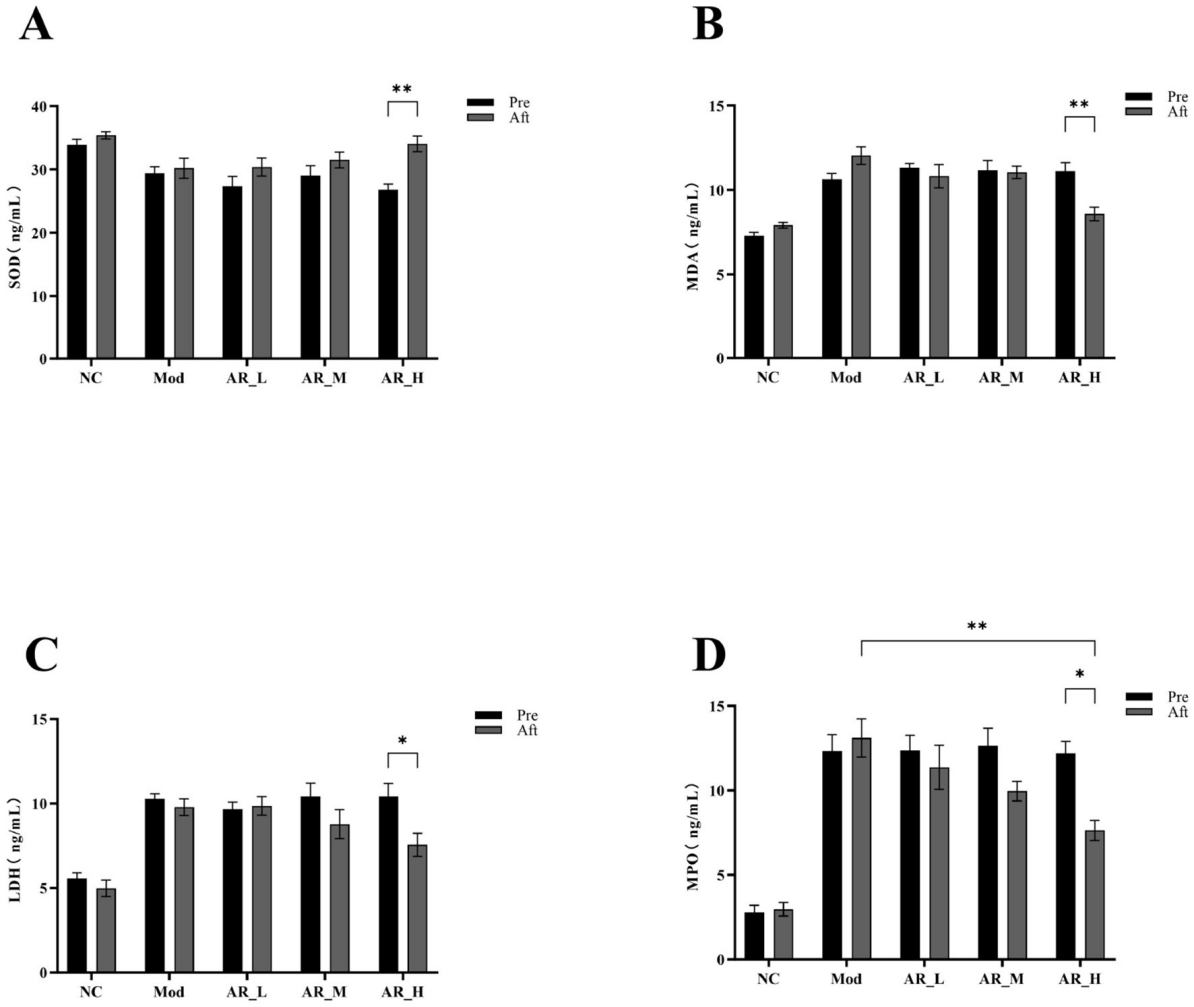
Changes of the serum oxidative stress markers. **(A)** SOD; **(B)** MDA; **(C)** LDH; **(D)** MPO. The differences between the two groups are indicated by (^*^), ^*^P < 0.05, ^**^P < 0.01.

Post-intervention analysis revealed significant increases in SOD activity (*P* < 0.01) and marked reductions in MDA (*P* < 0.01), LDH (*P* < 0.05), and MPO (*P* < 0.05) levels in the AR_H group. Notably, AR_H group MPO levels were significantly lower than MOD group (*P* < 0.01). No significant differences were observed between AR_M and AR_L groups (Figure 5). These findings demonstrate that Astragalus supplementation, particularly at high doses, significantly enhanced antioxidant capacity and alleviated oxidative stress, while lower doses exhibited moderate effects.

### 3.6 Effects of ARWD on immunoglobulins in SCBM cows

**Figure 6:**
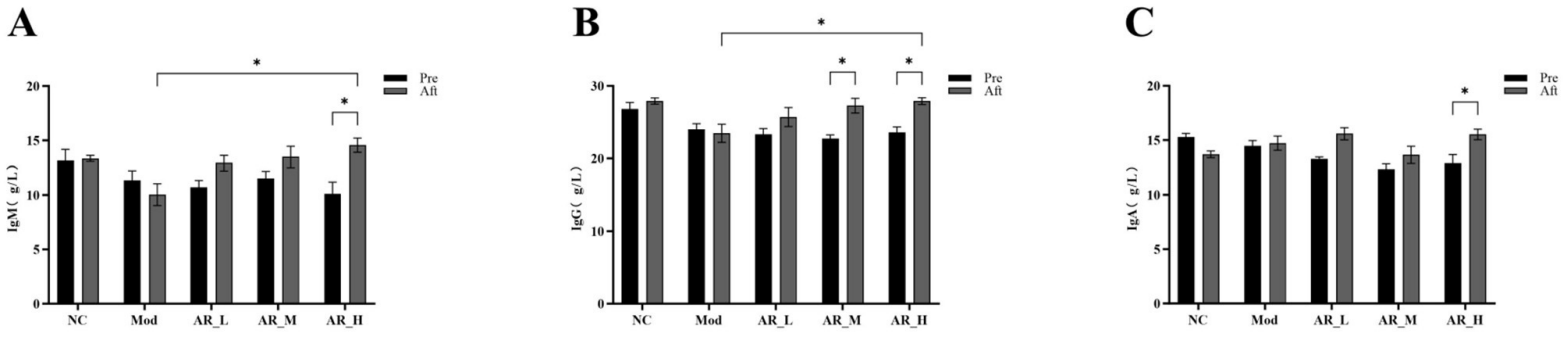
Changes of the serum Immunoglobulin. **(A)** IgM; **(B)** IgG; **(C)** IgA. The differences between the two groups are indicated by (^*^), ^*^P < 0.05.

ARWD exerted significant modulatory effects on the immunoglobulin profiles in experimental groups. In the AR_H group, post-treatment levels of IgA, IgG, and IgM were significantly elevated compared to pre-treatment (*P* < 0.05). Furthermore, AR_H exhibited marked increases in IgM and IgG vs. the MOD group (*P* < 0.05). In contrast, AR_M and AR_L groups demonstrated no significant alterations in immunoglobulin levels relative to MOD (*P* > 0.05) (Figure 6). These findings indicate that high-dose Astragalus supplementation enhanced immune function through immunoglobulin modulation, while medium- and low-dose groups showed marginal efficacy.

### 3.7 Effects of ARWD on rumen and gut microbiota in SCBM cows

#### 3.7.1 Rumen fluid microbiota sequencing

**Figure 7:**
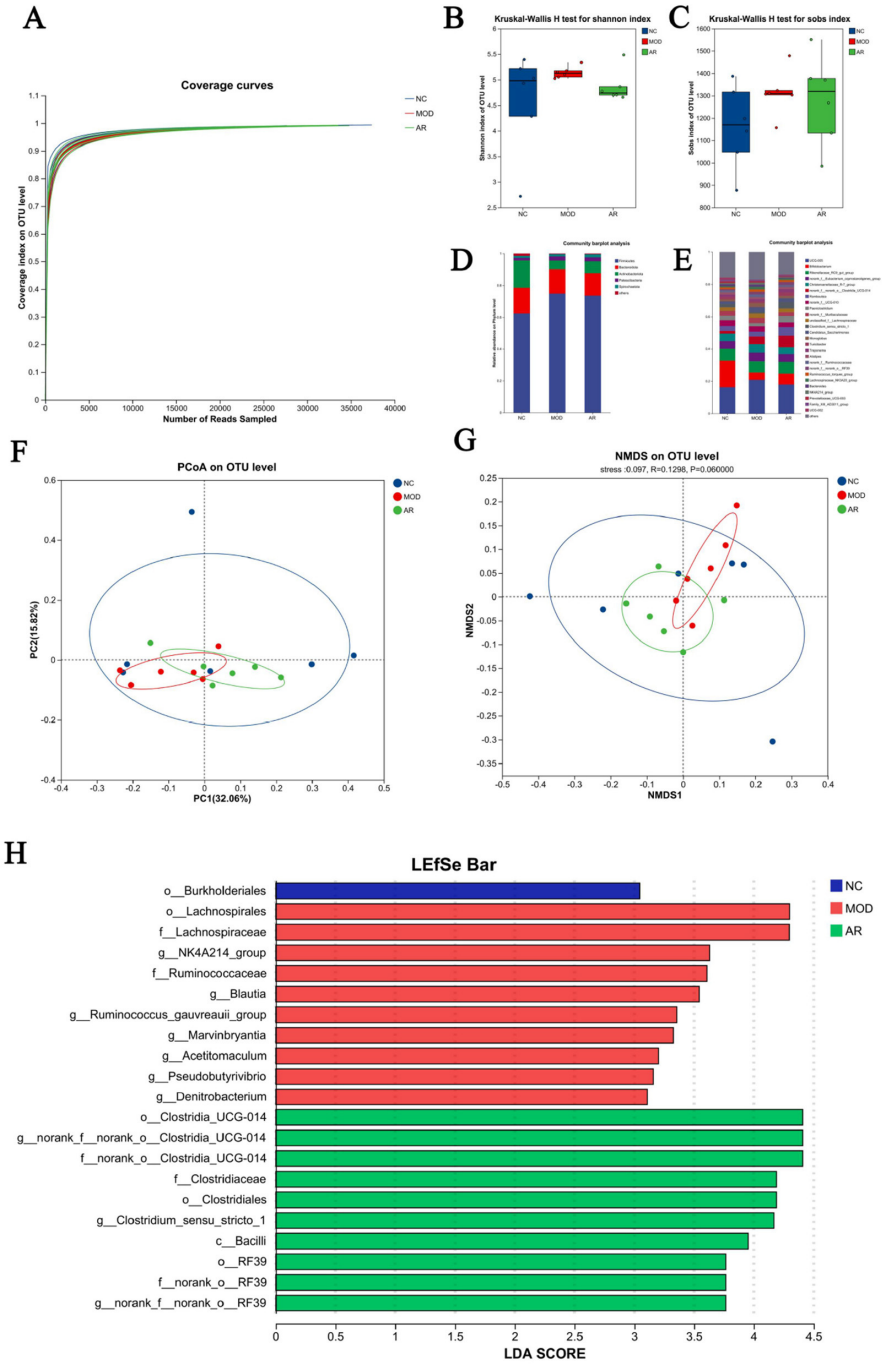
Diversity of the rumen bacterial flora. **(A)** Microbial species rarefaction curve; **(B)** Shannon; **(C)** Sobs; **(D)** phylum level; **(E)** genus levels; **(F)** PCoA; **(G)** NMDS; **(H)** LDA.

A total of 4, 323 valid 16S rRNA sequences were obtained from 18 rumen fluid samples. Clustering analysis of non-redundant sequences at 97% similarity threshold identified 2,058 operational taxonomic units (OTUs). Rarefaction curves approached saturation with increasing sequencing depth, indicating adequate sequencing coverage (Figure 7A).

#### 3.7.2 Rumen microbiota Alpha diversity analysis

No significant differences in Ace, Chao, Coverage, Shannon, Simpson, or Sobs indices were observed between Astragalus-treated and MOD groups (*P* > 0.05). However, downward trends in Shannon and Sobs indices were noted in the intervention group (Figures 7B, C). These results suggest potential modulatory effects of ARWD on rumen microbial richness and diversity in SCBM cows, though statistical significance was not achieved.

#### 3.7.3 Rumen fluid microbial composition at phylum and genus levels

Analysis of 18 rumen fluid samples revealed distinct microbial compositions at phylum and genus levels. Phylum-level composition identified 15 phyla. Firmicutes dominated across groups (62.5% in NC, 74.8% in MOD, 73.5% in AR_H group), followed by Bacteroidota (16.0%, 15.3%, 14.2%) and Actinobacteriota (17.2%, 5.4%, 7.4%). Minor phyla including Patescibacteria and Spirochaetota showed substantially lower abundances (< 2% collectively) (Figure 7D). Genus-level analysis detected 294 genera. Dominant genera in NC included *UCG-005* (16.2%), *Bifidobacterium* (16.6%), *Romboutsia* (3.1%), and *Paeniclostridium* (2.8%). MOD group exhibited altered profiles: *UCG-005* (20.8%), *Bifidobacterium* (4.6%), *Romboutsia* (3.0%), and *Paeniclostridium* (1.9%). Astragalus intervention induced notable compositional shifts vs. MOD group: increased abundances of *Bifidobacterium* (6.8%), *Romboutsia* (5.3%), and *Paeniclostridium* (4.0%), with reduced *UCG-005* (17.9%) (Figure 7E). These phylum- and genus-level microbial restructuring patterns suggest potential mechanistic links to Astragalus' therapeutic effects on SCBM in dairy cows.

#### 3.7.4 Rumen microbiota β-diversity analysis

Bray-Curtis distance-based PCoA and NMDS analyses revealed partial separation of microbial communities among groups. The AR_H group exhibited distinct clustering from the MOD group, with closer proximity to the NC group, suggesting partial restoration of rumen microbiota structure in SCBM cows (Figures 7F, G). However, limited effects were observed on fecal microbiota β-diversity indices.

#### 3.7.5 LEfSe analysis of rumen microbiota

LEfSe analysis with linear discriminant analysis (LDA) revealed significant microbial shifts between the MOD and AR_H groups (*P* < 0.05, Figure 7H). Compared to the MOD group, the AR_H group showed significant enrichment in beneficial genera such as *Clostridium_sensu_stricto_1* (*P* = 0.016), *Turicibacter* (*P* = 0.037), *Erysipelotrichaceae_UCG-008* (*P* = 0.028), and fiber-degrading taxa including *Cellulosilyticum* (*P* = 0.004) *Clostridium_sensu_stricto_6* (*P* = 0.036) *hoa5-07d05_gut_group* (*P* = 0.007). Conversely, *Blautia* (*P* = 0.006), *Ruminococcus_gauvreauii_group* (*P* = 0.016), *Roseburia* (*P* = 0.004) and other inflammation-associated genera, and *Brevibacillus* (*P* = 0.028), *Pseudobutyrivibrio* (*P* = 0.006), *Marvinbryantia* (*P* = 0.006) and other Potential pathobionts were significantly reduced. These microbiota alterations suggest that ARWD may alleviate SCBM by modulating microbial communities linked to immune regulation and metabolic balance.

#### 3.7.6 Fecal microbiota sequencing

**Figure 8:**
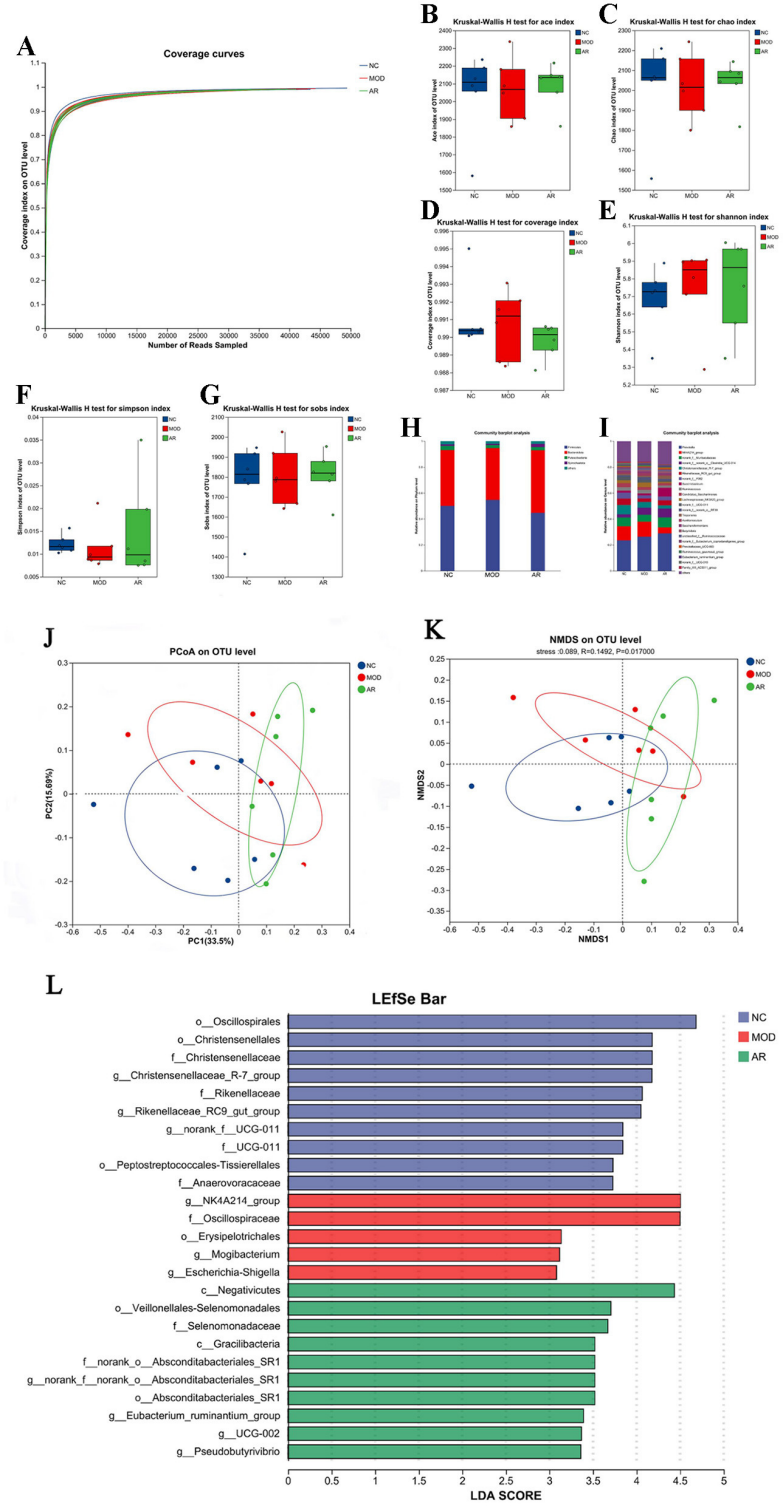
Diversity of the fecal microflora. **(A)** Feces microbial species rarefaction curve; **(B)** ACE; **(C)** Chao; **(D)** coverage; **(E)** Shannon; **(F)** Simpson; **(G)** Sobs;**(H)** phylum level; **(I)** genus levels; **(J)** NMDS; **(K)** PCoA; **(L)**LDA.

A total of 5,216 high-quality 16S rRNA sequences were obtained from 18 fecal samples. Clustering at 97% similarity threshold yielded 2, 290 OTUs. The rarefaction curve plateaued with increasing sequencing depth (Figure 8A), confirming adequate sampling coverage to capture microbial diversity.

#### 3.7.7 Fecal microbiota Alpha diversity

Alpha diversity indices (Ace, Chao, Shannon, Simpson, Sobs, and Coverage) showed increased trends in the AR_H group compared to MOD, though without statistical significance (*P* > 0.05, Figures 8B–G). This non-significant elevation in richness (Ace/Chao) and evenness (Shannon/Simpson) suggests a potential but modest modulatory effect of Astragalus intervention on microbial community structure in SCBM cows.

#### 3.7.8 Fecal microbial composition at phylum and genus levels

Taxonomic classification using the RDP classifier and Bayesian algorithm identified 19 phyla and 320 genera across 18 fecal samples. Phylum-level analysis revealed dominant taxa across groups: Firmicutes: 50.1% (NC), 54.7% (MOD), 44.7% (AR_H). Bacteroidota: 43.1% (NC), 39.95% (MOD), 48.25% (AR_H). Minor phyla: Patescibacteria (3.2%, 2.3%, 2.5%) and Spirochaetota (1.2%, 1.04%, 2.5%) (Figure 8H). Genus-level profiling demonstrated group-specific dominance: *NK4A214_group*: 10.94% (NC), 11.52% (MOD), 4.57% (AR_H). *Christensenellaceae_R-7_group*: 7.13% (NC), 4.49% (MOD), 2.5% (AR_H). Functional shifts: *Succiniclasticum* increased to 6.69% in AR_H (vs. 1.44% in MOD), while *Ruminococcus_gauvreauii_group* declined to 0.68% (vs. 1.27% in MOD) (Figure 8I). These results highlight distinct fecal microbiota restructuring at both taxonomic levels following Astragalus intervention in SCBM cows.

#### 3.7.9 Fecal microbiota Beta-diversity analysis

Beta-diversity analysis based on Bray-Curtis distance revealed partial compositional shifts among NC, MOD, and AR_H groups. PCoA and NMDS plots demonstrated distinct clustering of AR_H group from MOD, with a convergence trend toward CON (Figures 8J, K). These findings suggest *Astragalus* intervention partially restored microbial richness and diversity in SCBM cows, though its effects on β-diversity metrics remained statistically non-significant, indicating limited structural reorganization of the fecal microbiota community.

#### 3.7.10 LEfSe analysis of fecal microbiota across groups

LEfSe analysis with LDA identified significant microbial biomarkers between MOD and AR_H groups (*P* < 0.05, Figure 8L). Compared to MOD, the AR_H group exhibited enrichment of fiber-degrading genera (e.g., *Treponema, P* = 0.037; *Selenomonas, P* = 0.01) and metabolic regulators (*Anaerovibrio, P* = 0.025), alongside suppression of mastitis-associated taxa (*Ruminococcus_gauvreauii_group, P* = 0.037; *Corynebacterium, P* = 0.007). Notably, opportunistic pathogens (*Brevibacillus, P* = 0.002) and inflammation-linked genera (*Roseburia, P* = 0.037) were reduced. These findings highlight Astragalus-induced remodeling of gut microbiota, potentially mediating systemic anti-inflammatory effects via the gut-mammary axis in SCBM cows.

### 3.8 Identification of serum characteristic metabolites and analysis of related metabolic pathways in cows with SCBM

**Figure 9:**
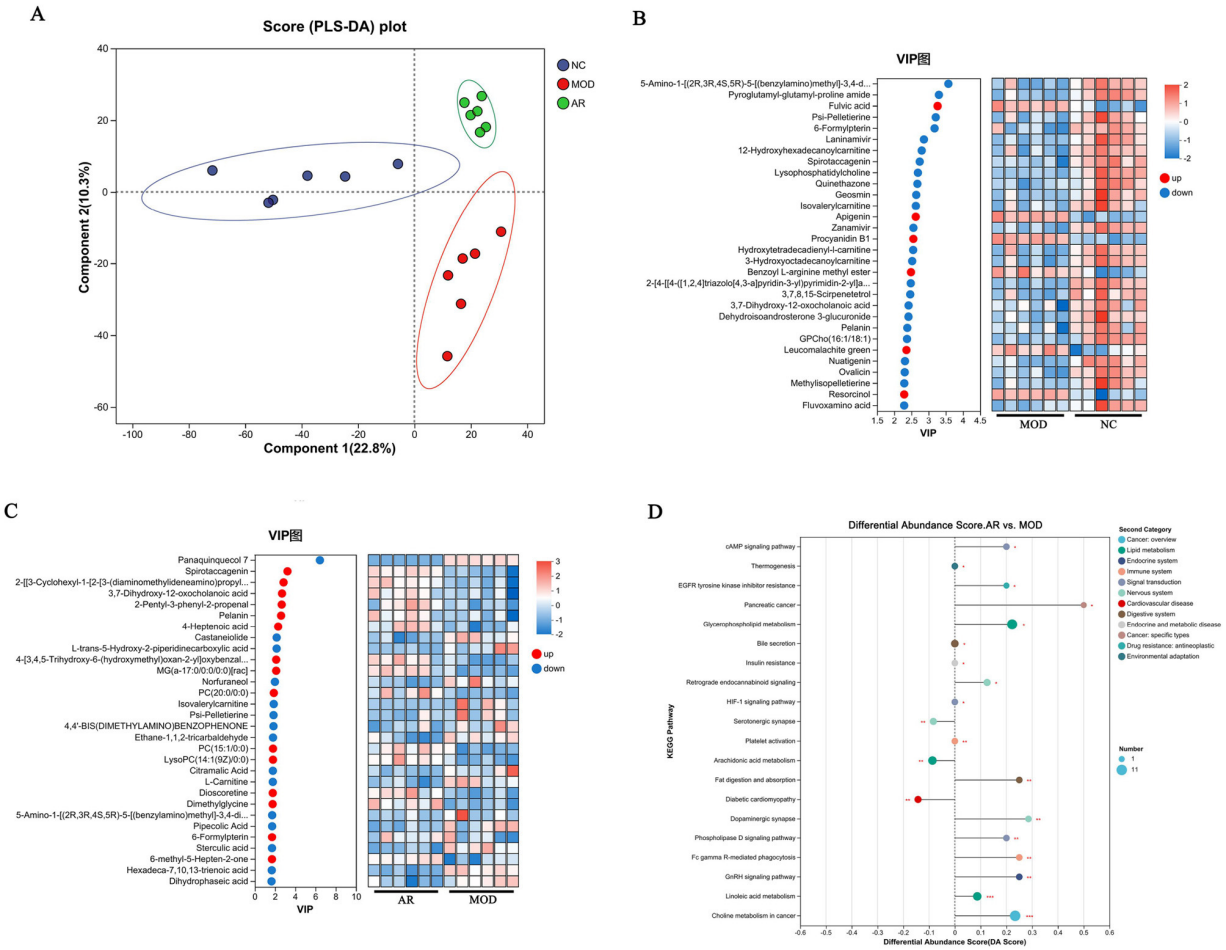
Effects of AR_H on the serum metabolites. **(A)** PLS-DA score plot; **(B)** heatmap of MOD vs. NC; **(C)** heatmap of AR vs. MOD; **(D)** KEGG pathway enrichment analysis.

Orthogonal partial least squares-discriminant analysis (OPLS-DA) was employed to investigate metabolic disparities. As shown in Figure 9A, score plots in both positive and negative ion modes revealed distinct clustering patterns among groups (NC, MOD, and AR_H), with tight intra-group sample aggregation, indicating significant intergroup differences (*P* < 0.05) and robust data reproducibility. Differential metabolites were screened using criteria of variable importance in projection (VIP) > 1.0 and *P* < 0.05, identifying 270 metabolites between MOD and NC groups and 198 metabolites between AR_H and MOD groups (Figures 9B, C). The OPLS-DA/PLS-DA models, validated by seven-fold cross-validation, highlighted metabolites critical for group classification via VIP analysis.

MOD vs. NC comparisons showed 24 significantly downregulated and 6 upregulated metabolites. In AR_H vs. MOD, 15 metabolites were downregulated and 15 upregulated. Notably, MOD exhibited marked reductions in *3, 7-dihydroxy-12-oxocholanoic acid, pelanin*, and *6-formylpterin* compared to NC (*P* < 0.05), while AR_H restored these metabolites to near-normal levels (*P* < 0.05). Pathway analysis identified five dysregulated pathways in MOD vs. NC: *linoleic acid metabolism* (*P* = 0.003)*, choline metabolism in cancer* (*P* = 0.007)*, retrograde endocannabinoid signaling* (*P* = 0.012)*, cAMP signaling pathway* (*P* = 0.018)*, and phospholipase D signaling* (*P* = 0.023). AR_H significantly restored these pathways (Figure 9D), suggesting its therapeutic role in modulating lipid-associated inflammation and cellular signaling cascades in SCBM.

### 3.9 Correlation analysis between serum metabolites and gastrointestinal microbiota

**Figure 10:**
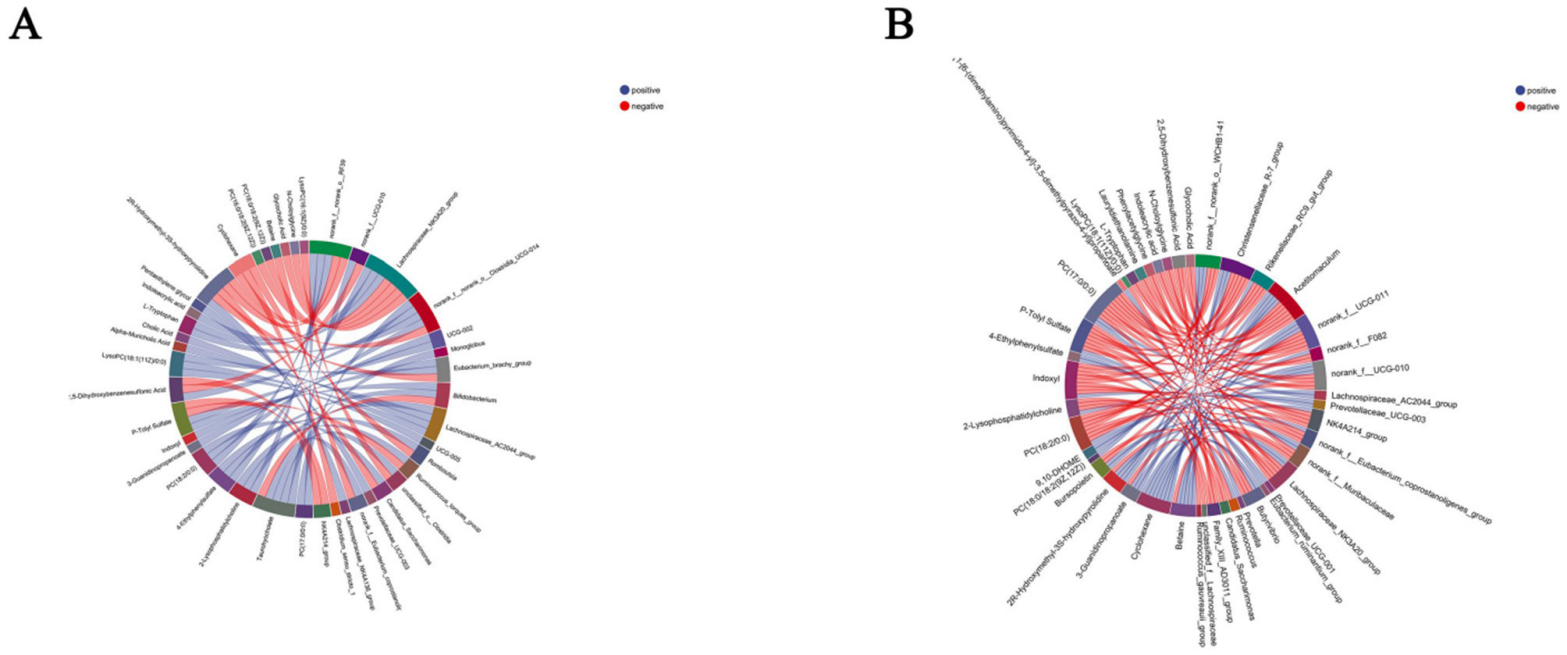
Correlation analysis results between serum differential metabolites and gastrointestinal microbiota. **(A)** Serum differential metabolites-rumen microbiota correlation; **(B)** serum differential metabolites-intestinal microbiota correlation. Red and blue lines indicate positive and negative correlations, respectively.

In this study, seven differential metabolites significantly associated with rumen microbiota (*R* > 0.5, *P* < 0.05) were identified (Figure 10A). Among them, betaine exhibited a positive correlation with *norank_f__norank_o__WCHB1-41*, cyclohexane with *Christensenellaceae_R-7_group*, and 3-guanidinopropanoate with *Rikenellaceae_RC9_gut_group*, suggesting that these metabolites may promote or be linked to the abundance or activity of these bacterial taxa. Conversely, PC (17:0/0:0) and indoxyl showed negative correlations with *Christensenellaceae_R-7_group*, lauryldiet with *norank_f__F082*, and betaine with *norank_f__Muribaculaceae*, indicating potential inhibitory effects on the growth or function of these microbial populations. These findings highlight the complex interactions between distinct metabolites and rumen microbiota, which may play a pivotal role in modulating host rumen microbial communities and providing a foundation for further exploration of underlying mechanisms.

Four differential metabolites displayed strong correlations with fecal microbiota (*R* > 0.5, *P* < 0.05) (Figure 10B). Specifically, PC (17:0/0:0) was positively correlated with *norank_f__norank_o__RF39*, whereas p-tolyl sulfate showed a negative correlation with *NK4A214_group*, taurohyocholate with *Bifidobacterium*, and lysoPC (16:1(9Z)/0:0) with *unclassified_c__Clostridia*. These results suggest that these metabolites may influence the composition and activity of fecal microbiota, underscoring the potential role in regulating the fecal microbial dynamics.

### 3.10 Correlation analysis between gastrointestinal microbiota and SCC, milk yield, milk composition, and serum parameters

Spearman correlation analysis was performed to evaluate the associations between the relative abundance of gastrointestinal microbiota at the genus level and 18 metrics, including SCC, milk yield, milk composition, and serum parameters.

**Figure 11:**
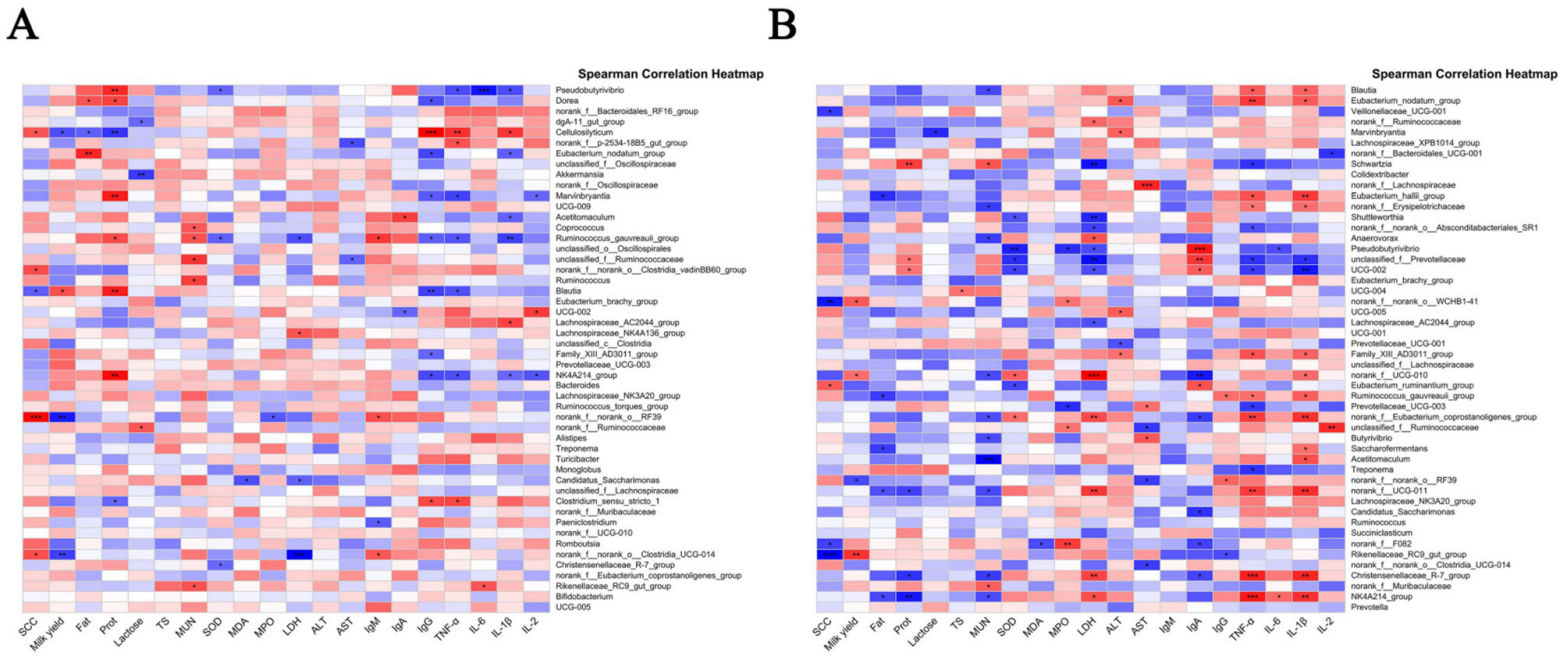
Correlation analysis results between gastrointestinal microbiota and clinical parameters. **(A)** Correlation between rumen microbiota and clinical parameters; **(B)** correlation between intestinal microbiota and clinical parameters. Red and blue colors indicate positive and negative correlations, respectively.

As illustrated in Figure 11A, 29 OTUs displayed significant correlations with at least one metric. Notably, *Acetitomaculum, Ruminococcus_gauvreauii_group, Pseudobutyrivibrio, norank_f__norank_o__RF39*, and *Monoglobus* in the rumen showed significant positive correlations with SCC, whereas *UCG-014* exhibited a negative correlation with SCC (*P* < 0.05), suggesting its potential protective role. Conversely, *Pseudobutyrivibrio* and *Ruminococcus_gauvreauii_group* were negatively correlated with milk yield, indicating detrimental associations with lactation performance.

Key correlations with inflammatory markers were also observed: *Lachnospiraceae_NK4A136_group, Balautia, unclassified_f_Lachnospiraceae*, and *Dorea* displayed positive correlations with TNF-α, while *Balautia, NK4A214_group, Bacteroides*, and *unclassified_f_Oscillospirales* were positively correlated with IL-6 (*P* < 0.05), highlighting their involvement in inflammatory responses. Intriguingly, *Acetitomaculum* and *Ruminococcus_gauvreauii_group* showed positive correlations with IL-1β, LDH, and MPO—markers of inflammation and cellular damage—but negative correlations with SOD, lactose, IgG, and milk fat (*P* < 0.05), linking these genera to oxidative stress and milk quality deterioration. These findings reveal significant associations between specific rumen microbial taxa and critical clinical/biochemical parameters, identifying potential microbial targets for improving host health and productivity.

In the fecal microbiota (Figure 11B), *Eubacterium_ruminantium_group* was positively correlated with SCC, whereas *Rikenellaceae_RC9_gut_group, norank_f__norank_o__WCHB1-41, norank_f__F082*, and *Veillonellaceae_UCG-001* exhibited negative correlations with SCC (*P* < 0.05), implying potential anti-inflammatory properties. Notably, *norank_f__UGG-010, norank_f__norank_o__WCHB1-41*, and *Rikenellaceae_RC9_gut_group* demonstrated positive correlations with milk yield, highlighting their potential role in yield enhancement. *Norank_f__norank_o__RF39* and *Eubacterium_ruminantium_group* were positively correlated with LDH, linking them to tissue damage and inflammatory states. Additionally, genera such as *Eubacterium_nodatum_group, Eubacterium_hallii_group, norank_f__Eubacterium_coprostanoligenes_group, norank_f__UCG-011, Christensenellaceae_R-7_group*, and *NK4A214_group* showed positive correlations with TNF-α (*P* < 0.05), emphasizing their participation in systemic inflammation.

The therapeutic efficacy of ARWD in SCBM might largely stem from its ability to modulate gastrointestinal microbiota, particularly by influencing microbial taxa associated with inflammation, milk production, and systemic health. These results underscore the pivotal role of gastrointestinal microbiota in both the pathophysiology and treatment of SCBM.

### 3.11 Correlation analysis between serum metabolites and SCC, milk yield, milk composition, and serum parameters

**Figure 12:**
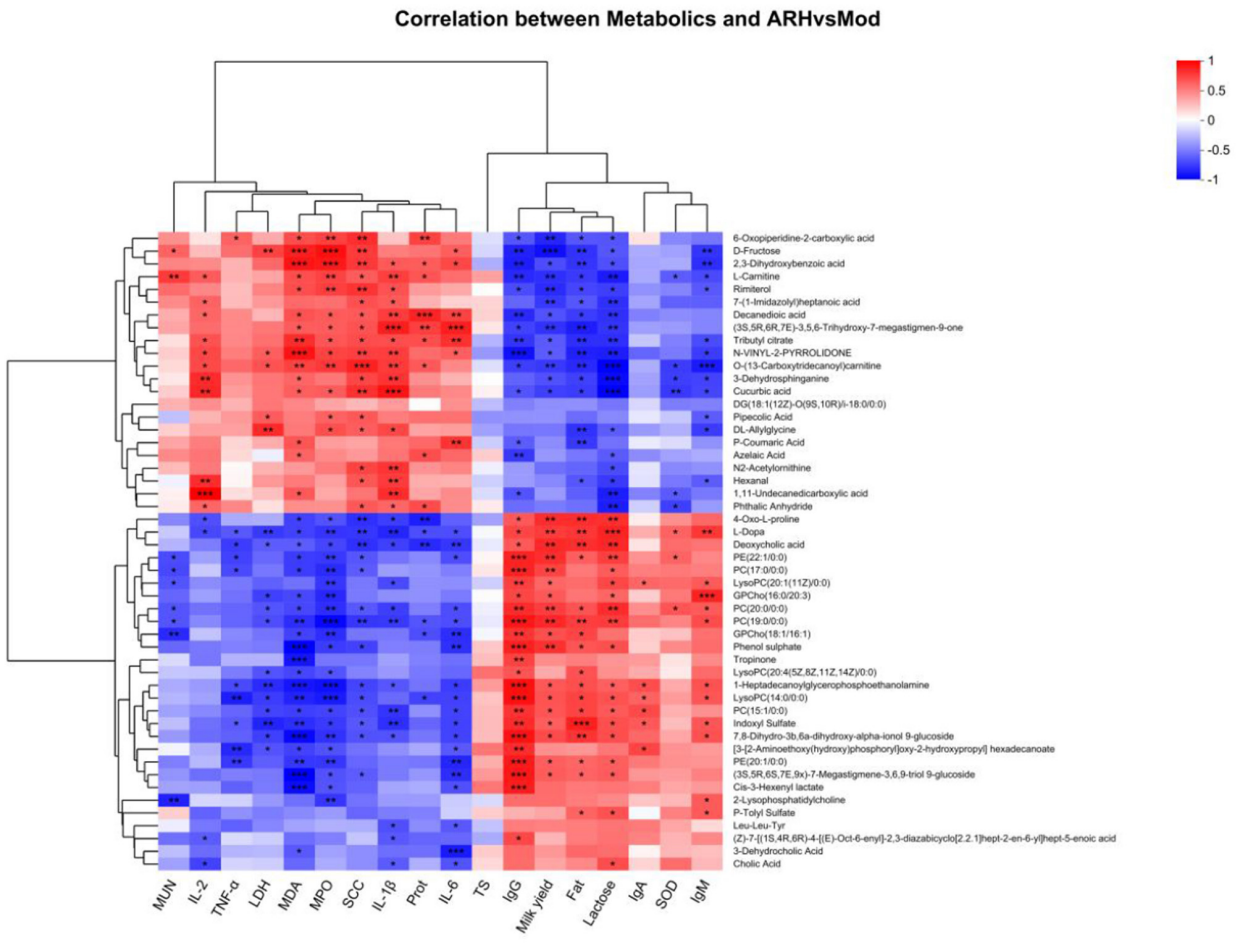
Correlation analysis results between serum metabolites and clinical parameters. Red and green colors indicate positive and negative correlations, respectively; *P* values are denoted as ^*^ < 0.05, ^**^ < 0.01, ^***^ < 0.001.

Correlation analysis between serum metabolites (AR_H vs. MOD) and clinical indicators, milk composition, and serum parameters (Figure 12) revealed significant changes in metabolite levels associated with SCC, serum parameters, and milk composition following ARWD intervention. Notably, serum *D-fructose* exhibited positive correlations with SCC, LDH, and MDA, but negative correlations with milk yield, fat, and lactose (*P* < 0.05), suggesting its association with inflammation and impaired milk quality. Furthermore, metabolites such as *6-oxopiperidine-2-carboxylic acid, 2, 3-dihydroxybenzoic acid, L-carnitine, decanedioic acid*, and *(3S, 5R, 6R, 7E)-3, 5, 6-trihydroxy-7-megastigmen-9-one* showed significant positive correlations with SCC, MDA, and MPO, but negative correlations with milk yield, fat, lactose, and IgG (*P* < 0.05), implicating their roles in inflammatory responses, oxidative stress, and immune dysfunction. Conversely, *PE (20:1/0:0)* demonstrated a negative correlation with TNF-α and IL-6 (*P* < 0.05), highlighting the potential anti-inflammatory properties.

These results indicate that AR_H effectively modulates serum metabolites, reduces SCC, alleviates inflammation and oxidative stress, and improves milk yield and immune function. Taken together, these results highlight *Astragali Radix* as a promising traditional herbal formulation for the treatment of SCBM in dairy cows.

## 4 Discussion

SCBM in dairy cows remains a critical health challenge requiring urgent resolution in modern livestock farming. With increasing antibiotic resistance and evolving veterinary drug regulations, there is a pressing need to develop greener and healthier alternatives (15). In this study, untargeted metabolomic was employed to analyze the bioactive components of ARWD and integrated serum metabolomics with 16S rRNA sequencing were used to elucidate the therapeutic mechanism on SCBM of ARWD.

To clarify the bioactive basis of ARWD in treating bovine mastitis, LC-MS/MS analysis identified nine active compounds, including *Astragalosides* III, VI, and IV, ononin, formononetin, and their derivatives. These compounds were found to exhibit anti-inflammatory, antioxidant, and immunomodulatory properties. Notably, *Astragaloside* III demonstrated significant anti-inflammatory activity by reducing inflammatory responses and accumulating in immune organs such as the thymus and spleen, highlighting its immunomodulatory potential (16). Furthermore, *Astragaloside polysaccharides* and *Astragaloside* IV markedly suppressed the expression of pro-inflammatory cytokines and apoptosis in lipopolysaccharide (LPS)-induced bovine mammary epithelial cell models, underscoring their pivotal role in mitigating mastitis (17). Other components, such as *ononin* and *formononetin*, also showed therapeutic promise. *Ononin* reduced ROS generation and inhibited pro-inflammatory factors, suggesting broad applications in anti-inflammatory and anticancer therapies (18). Similarly, *formononetin* alleviated LPS-induced mastitis symptoms by enhancing the integrity of the lactation barrier and suppressing AhR-Src signaling pathway activation (19). Additionally, bioactin A demonstrated robust anti-inflammatory and immune-enhancing properties, further supporting its development as a therapeutic agent for mastitis (20).

SCC serves as a critical biomarker for assessing udder health in dairy cows, where significant elevation in SCC levels typically indicates mastitis. The increase in SCC is primarily attributed to immune cell infiltration into mammary tissues, triggering inflammatory responses. Studies have shown that mastitis pathogenesis involves LPS and pathogenic microorganisms stimulating the release of pro-inflammatory cytokines such as IL-1β and TNF-α, leading to inflammatory damage in mammary tissues and marked increases in SCC (21, 22). Elevated SCC levels are often associated with impaired mammary barrier function, resulting in sustained pathogenic stimulation and exacerbated inflammation. Clinical studies further reveal significant differences in lactation performance between cows with varying SCC levels (23). In healthy cows, milk proteins predominantly comprise casein, whey proteins, and minor non-protein nitrogen components. During mastitis, inflammatory mediators disrupt mammary epithelial cell function and increase barrier permeability, causing an imbalance in protein composition and reduced total protein content. Notably, the proportion of casein declines significantly, while concentrations of whey proteins such as lactoferrin and lactoglobulin rise substantially. These shifts likely reflect the activation of mammary immune defenses, which enhance the secretion of whey proteins, particularly antimicrobial proteins, to combat infection (24, 25).

Mastitis-induced changes in osmotic gradients facilitate the leakage of plasma proteins such as albumin and fibrinogen into milk, further altering milk protein composition and potentially compromising dairy processing quality. Studies also indicate that cows with elevated SCC levels typically have lower milk protein content, which is associated with the metabolic burden of inflammation as well as physical damage and functional decline in mammary tissues (25).

The development of bovine mastitis is closely associated with the overexpression of pro-inflammatory cytokines, particularly IL-1β, IL-6, and TNF-α, which play pivotal roles in inflammatory cascades (26). IL-1β acts as a critical initiator of inflammatory responses by activating NF-κB and MAPK signaling pathways, thereby inducing the release of other pro-inflammatory cytokines and significantly enhancing neutrophil migration into mammary tissues, exacerbating tissue damage (27). Elevated IL-1β levels in milk from mastitic cows correlate positively with increased SCC (26). IL-6, a key mediator of acute-phase responses, enhances antimicrobial defenses by stimulating lactoferrin and C-reactive protein production. However, its role in increasing vascular permeability may also promote inflammatory dissemination (28). Prolonged IL-6 overexpression further impairs mammary barrier function and is strongly associated with reduced lactose secretion. TNF-α, another central regulator of pro-inflammatory responses, induces apoptosis and oxidative stress, aggravating mammary tissue lesions (29). Our findings demonstrate that ARWD significantly reduces IL-1β, IL-6, and TNF-α expression levels in milk from mastitic cows. These results suggest that ARWD alleviates mammary inflammation and tissue damage by disrupting cytokine-triggered inflammatory cascades. Consistent with this, Khan et al. (30) reported that certain natural plant-derived bioactive compounds regulate mastitis-associated cytokines. By suppressing the overexpression of pro-inflammatory cytokines, ARWD effectively mitigates mastitis symptoms, preserves mammary tissue integrity, and improves lactation performance in dairy cows.

The onset of bovine mastitis is accompanied by exacerbated oxidative stress, characterized by dysregulated levels of oxidative biomarkers such as MPO, LDH, SOD, and MDA. MPO, a key oxidative enzyme released by neutrophils, reflects the intensity of inflammatory responses and neutrophil hyperactivation. Elevated MPO activity is recognized as a marker of inflammatory severity in mastitic cows (31, 32). LDH, an indicator of cellular damage, increases significantly during mastitis due to inflammation and necrosis in mammary tissues, signifying impaired tissue metabolism and compromised mammary barrier integrity (33). Concurrently, reduced SOD activity—a critical antioxidant enzyme counteracting oxidative stress—leads to free radical accumulation, aggravating tissue damage. MDA, a lipid peroxidation byproduct, exhibits elevated concentrations indicative of oxidative membrane damage (34). Our results demonstrate that ARWD significantly reduced MPO and LDH activities in mastitis models while elevating SOD levels and reducing MDA concentrations. These findings highlight ARWD's efficacy in mitigating oxidative stress-induced tissue damage, underscoring its potential as a therapeutic agent for bovine mastitis.

The pathogenesis of bovine mastitis is accompanied by immune system activation (35), with immunoglobulins IgM, IgG, and IgA playing critical roles in mammary immune defense. During mastitis, LPS translocation from the rumen to the bloodstream enhances pro-inflammatory cytokine release and elevates serum immunoglobulin levels (36). IgM, the primary antibody in the initial immune response, rapidly recognizes mastitis-associated pathogens and facilitates pathogen clearance by activating the complement system. IgG, the predominant antibody in bovine mammary immunity, provides protection by neutralizing pathogen toxins and enhancing phagocyte functionality. The marked increase in milk IgG levels during mastitis reflects sustained immune responses to mammary infections (28). Additionally, IgA, a key component of local mucosal immunity, prevents pathogen adhesion to mammary epithelial cells, thereby reducing tissue damage. Studies indicate that elevated IgA levels in bovine milk strengthen local immune barriers and enhance protective functions (28). In this study, ARWD significantly elevated serum IgM, IgG, and IgA levels in mastitic cows, indicating its dual role in augmenting systemic primary immune responses and suppressing inflammatory progression via improved local mammary immunity. These findings align with reports by Khan et al. (30), who demonstrated that certain herbal components effectively upregulate immunoglobulin expression linked to bovine mammary immunity. Collectively, this underscores the protective effects and theoretical rationale for using traditional Chinese medicine in mastitis management.

The rumen microbiota plays a pivotal role in both the development and therapeutic management of bovine mastitis by modulating systemic immune functions during inflammatory responses (29, 37). Our study revealed significant shifts in microbial diversity within the rumen and intestines of mastitic cows, with ARWD intervention promoting a restorative trend in microbiota composition. Notably, key genera such as *Turicibacter, Cellulosilyticum, Brevibacillus, Roseburia*, and *Saccharofermentans* were implicated in these dynamics.

*Turicibacter* is a strictly anaerobic, Gram-positive, rod-shaped bacterium typically abundant in the gut and rumen of healthy animals. It critically maintains microbial balance, supports host metabolic health, and regulates immune functions (38). *Cellulosilyticum* is a cellulolytic genus essential for degrading plant fibers in the rumen, producing volatile fatty acids (VFAs) vital for energy metabolism. Mastitis-induced reductions in its abundance impair energy homeostasis and compromise immune defenses (39). *Brevibacillus* is a Gram-positive, spore-forming, thermotolerant genus within the Bacillaceae family, exhibiting aerobic/facultative anaerobic traits. Brevibacillus strains demonstrate resistance to 67% of tested antibiotics, suggesting potential interference with mastitis treatment (40). *Roseburia* is a strictly anaerobic, Gram-positive genus prevalent in mammalian intestines. Zhao et al. (41) reported that citrus flavonoid extracts reduce its abundance, improving inflammatory and immune-metabolic functions in cows. *Saccharofermentans* is an acid-producing bacterium critical for mammary barrier integrity. Its depletion correlates with metabolic dysregulation and reduced milk protein levels in lactating cows (42). Our findings demonstrate that ARWD significantly restored the abundance of beneficial rumen microbiota, particularly *Turicibacter* and *Cellulosilyticum*. These results suggest that ARWD alleviates mastitis by rebalancing rumen microbiota and enhancing metabolic functions, thereby mitigating inflammation and supporting mammary health.

The onset of bovine mastitis is frequently accompanied by significant gut microbiota disruption, which not only alters microbial diversity but also directly impacts host immunometabolic and inflammatory responses. Our study identified marked changes in the abundance of *Roseburia, Treponema, Selenomonas, Prevotellaceae_UCG-004, Corynebacterium, Staphylococcus, Microbacterium*, and *Eubacterium_nodatum_group* in the gut microbiota of mastitic cows. *Roseburia*, a primary producer of short-chain fatty acids (SCFAs) via butyrate metabolism, plays a pivotal role in maintaining intestinal barrier integrity and suppressing systemic inflammation. Its depletion in mastitic cows is strongly associated with exacerbated immune dysfunction (41). Conversely, *Treponema*, a Gram-negative spirochete linked to chronic inflammation and tissue damage, exhibited elevated abundance, potentially exacerbating mastitis through lipopolysaccharide (LPS)-mediated immune hyperactivation (43). Similarly, increased *Selenomonas* (a Gram-negative genus involved in metabolic regulation) abundance may disrupt metabolic homeostasis and inflammatory control, aggravating mammary tissue injury (44). *Prevotellaceae_UCG-004*, enriched in high-fiber diets, was modulated by dietary calcium propionate to improve energy metabolism and hypocalcemia (45). Pathogenic roles were observed for Corynebacterium (46) and *Staphylococcus/Microbacterium* (47) with Astragalus supplementation specifically inhibiting *Microbacterium* to exert anti-inflammatory effects. Notably, *Eubacterium_nodatum_group*, a Gram-positive anaerobic commensal linked to metabolic regulation, showed reduced abundance in *Astragalus*-treated buffalo with mastitis, suggesting its role in microbiota-driven therapeutic modulation (39). These findings underscore the potential of *Astragalus* to alleviate mastitis by restoring gut microbiota balance and targeting pathogenic taxa, highlighting its dual role in metabolic and immune regulation.

ARWD significantly increased the abundance of the gut microbiota genus *Roseburia* while reducing *Treponema* and *Selenomonas* levels, suggesting its ability to alleviate mastitis by restoring gut microbial equilibrium, suppressing pro-inflammatory bacteria, and improving systemic immune-metabolic homeostasis. These findings highlight ARWD potential to mitigate clinical manifestations of bovine mastitis.

Serum metabolomics identified four key differential metabolites in cows with SCBM: *spirotaccagenin, 3, 7-dihydroxy-12-oxocholanoic acid, pelanin*, and *6-formylpterin*. These metabolites were linked to dysregulated pathways, including *linoleic acid metabolism, choline metabolism in cancer, retrograde endocannabinoid signaling, cAMP signaling*, and *phospholipase D signaling*, which collectively influence inflammatory responses, oxidative stress, and immune regulation. Spirotaccagenin is a steroidal compound hypothesized to exert anti-inflammatory and immunostimulatory effects via downstream steroidal glycosides, which modulate cell proliferation and antimicrobial activity (48, 49). 3,7-Dihydroxy-12-oxocholanoic acid is a bile acid derivative critical for lipid digestion and absorption, with potential roles in gut microbiota modulation and immune function (50). Pelanin is an anthocyanin derivative renowned for its antioxidant and anti-inflammatory properties, attenuating chronic inflammation via inhibition of NF-κB and STAT1/3 signaling pathways (51). 6-Formylpterin: Suppresses lipopolysaccharide (LPS)-induced nitric oxide (NO) production in macrophages, demonstrating anti-inflammatory potential (52). Pathway analysis revealed that linoleic acid metabolism—a dual modulator of pro- and anti-inflammatory responses through its role as a precursor for arachidonic acid—enhances bacterial clearance in macrophages, suggesting lipid-mediated immune defense (53). Similarly, the *phospholipase D* signaling pathway regulates cellular stress and inflammation during mastitis by modulating secondary messengers involved in proliferation and anti-apoptosis (54, 55). ARWD significantly elevated these metabolites and restored pathway activity, particularly in *linoleic acid* metabolism, thereby enhancing anti-inflammatory and antioxidant capacity. These findings elucidate ARWD molecular mechanisms in SCBM management, emphasizing its dual role in microbiota restoration and metabolic reprogramming to combat inflammation and oxidative stress.

## 5 Conclusion

ARWD effectively mitigates SCBM by modulating rumen-gut microbiota interactions and regulating linoleic acid metabolism and phospholipase D signaling. This intervention significantly reduced pro-inflammatory cytokines (IL-1β, TNF-α), enhanced immunoglobulins (IgM/IgG/IgA), and improved antioxidant capacity, achieving dual therapeutic benefits of lowered somatic cell counts and increased milk yield. These findings highlight ARWD's multi-target anti-inflammatory and antioxidant mechanisms, offering a sustainable alternative to antibiotics for mastitis management.

## Data Availability

The data presented in the study is deposited in the https://www.ncbi.nlm.nih.gov/ repository, accession number: SRP589200 and SRP589088.
